# Explanatory Factors of Vaccination Dropout Among Children Aged 12 to 23 Months in the Kikula Health Zone, Democratic Republic of Congo: Cross-Sectional Analytical Study

**DOI:** 10.2196/88879

**Published:** 2026-03-31

**Authors:** Blaise Musoya Mumba, Fiston Ilunga Mbayo, Pacifique Kanku Wa Ilunga, Hermann Tamubango Kitoko, Pascal Geri Madragule, Jean Nyandwe Kyloka

**Affiliations:** 1 School of Public Health, University of Kinshasa, 11850, Kinshasa, Democratic Republic of the Congo, 243 817689233, 243 995716784; 2 Department of Public Health, Malemba Nkulu General Reference Hospital, Malemba Nkulu, Haut-Lomami, Democratic Republic of the Congo; 3 School of Public Health, University of Malemba Nkulu, P.O. Box 365, Malemba Nkulu, Haut-Lomami, Democratic Republic of the Congo, +243817689233; 4 School of Public Health, Faculty of Medicine, University of Kamina, Kamina, Haut-Lomami, Democratic Republic of the Congo; 5 Department of Public Health, Likasi Institute of Advanced Medical Techniques, Likasi, Haut-Katanga, Democratic Republic of the Congo

**Keywords:** vaccination dropout, immunization coverage, child health, Democratic Republic of Congo, maternal knowledge, health system factors

## Abstract

**Background:**

Vaccination is among the most effective public health interventions to reduce childhood morbidity and mortality. Despite World Health Organization recommendations, global immunization coverage has declined in recent years, with the COVID-19 pandemic causing the largest sustained backslide in routine immunization in 3 decades. In the Democratic Republic of Congo (DRC), full immunization coverage remains below 50%, hindered by inequities, supply shortages, and financing delays. In the Kikula Health Zone, administrative reports suggest coverage exceeding 100%, yet independent surveys consistently reveal low completion and high dropout rates between Bacillus Calmette-Guérin (BCG) and measles vaccines. No previous study has specifically examined determinants of dropping out in this setting.

**Objective:**

This study assessed the prevalence and determinants of vaccination dropout between BCG and measles vaccines among children aged 12 to 23 months in the Kikula Health Zone, Likasi, DRC.

**Methods:**

An analytical cross-sectional survey was conducted from April 22 to May 22, 2025, using 3-stage cluster sampling to recruit 300 mother-child pairs. Vaccination status was verified using cards; for children without documentation, caregiver recall and health facility registers were used to minimize misclassification. Structured questionnaires captured sociodemographic data, child characteristics, maternal knowledge, perceptions of services, and health system access. Dropout was defined as receipt of BCG but not the measles vaccine. Bivariate associations were tested using chi-square tests, and multivariate logistic regression identified independent predictors, with robust SEs to account for clustering. Ethics approval was obtained from the University of Kinshasa School of Public Health.

**Results:**

Among 300 children, 115 (38.3%) had dropped out between BCG and measles vaccination, while 185 (61.7%) completed the schedule. Possession of a vaccination card was the strongest predictor: children without a card had 30-fold higher odds of dropout (adjusted odds ratio 30.9, 95% CI 11.6 82.0; *P*<.001). Other factors associated with dropout in bivariate analysis included shorter residence duration (≤5 y), lower maternal education, and nonuse of child health services, although these lost significance in multivariate models. Maternal knowledge gaps were notable: 169 (56.3%) did not know their child’s vaccination status and 148 (49.3%) expressed fear of side effects. Service perceptions were generally positive (participants reporting good reception: n=294, 98%), but 108 (36%) experienced waiting times of 1 to 2 hours. The exclusion of undocumented children likely led to underestimation of dropout prevalence.

**Conclusions:**

Vaccination dropout between BCG and measles remains high in the Kikula Health Zone, driven primarily by lack of vaccination cards and maternal knowledge gaps. Administrative coverage data (>100%) mask substantial dropout, underscoring the need for improved documentation, maternal education, and targeted outreach. Programmatic implications include strengthening card management, deploying mobile vaccination units, and enhancing community reminders. Findings highlight the importance of addressing both demand and supply-side barriers to reduce dropout and improve equity in immunization coverage in the DRC.

## Introduction

Vaccination is recognized as one of the most effective public health strategies for reducing childhood morbidity and mortality. The World Health Organization (WHO) recommends that all children receive vaccines against tuberculosis, diphtheria, pertussis, tetanus, poliomyelitis, measles, hepatitis B, *Haemophilus influenzae* type b, pneumococcus, rubella, and yellow fever before their first birthday in endemic countries [1]. Despite these recommendations, global immunization coverage has declined in recent years. Between 2019 and 2021, coverage with the third dose of the diphtheria-tetanus-pertussis vaccine (DTP3) fell from 86% to 81%, leaving 25 million children without full protection, including 18.2 million “zero-dose” children [2]. Similar declines were observed for the first dose of measles-containing vaccine (MCV1), which dropped from 85% to 81% [2]. The COVID-19 pandemic contributed to the largest sustained backslide in routine immunization in 3 decades, disproportionately affecting low- and middle-income countries [3,4].

In the WHO African Region, progress has remained limited. DTP3 coverage increased only modestly from 70% to 74% between 2013 and 2019, while MCV1 coverage declined slightly from 70% to 69% [1]. Updated assessments from 2023 confirm that Africa continues to account for the highest proportion of zero-dose children globally, driven by persistent inequities, service disruptions, and health system weaknesses [5]. In 2021, an estimated 25 million African children were not vaccinated, including 734,000 in the Democratic Republic of Congo (DRC) [1].

In the DRC, the Expanded Programme on Immunization aims to achieve ≥90% coverage for all antigens. The national immunization schedule recommends Bacillus Calmette-Guérin (BCG) at birth; 3 doses of DTP-HepB-Hib at 6, 10, and 14 weeks; and oral polio vaccine, pneumococcal conjugate vaccine, rotavirus vaccine, and measles vaccine at 9 months. However, full immunization coverage remains below 50%. The 2017‐2018 Multiple Indicator Cluster Survey (MICS) reported that only 35% of children aged 12 to 23 months were fully vaccinated, while 20% had received no vaccines at all [6,7]. Financing delays, recurrent supply shortages, and geographic inequities are major contributors to low coverage [6,7]. Recent multicountry analyses in sub-Saharan Africa confirm that maternal education, socioeconomic status, and health system accessibility remain key determinants of incomplete immunization [8,9].

In the Haut-Katanga province, administrative data suggested that 88.3% of children aged 0 to 11 months were fully vaccinated in 2021. Yet, vaccine-preventable diseases persisted: 1347 measles cases (including 349 zero-dose children), 34 acute flaccid paralysis cases, 101 meningitis cases, 14 neonatal tetanus cases, and 74 cholera cases. The BCG-measles dropout rate reached 11.4%, representing 35,680 children who did not complete their vaccination schedule [10]. In the Kikula Health Zone, administrative coverage was reported at 113.7% in 2022, but independent surveys conducted between 2020 and 2023 revealed that only 26% of children aged 12 to 23 months were fully vaccinated, with no improvement over time [11]. The validated dropout rate in this study was 38.3%, replacing earlier contextual estimates of 26% from prior surveys. This discrepancy between administrative data and field-based findings highlights structural, social, and health system factors influencing vaccination dropout.

No prior study has specifically examined the determinants of vaccination dropout in the Kikula Health Zone, representing a critical evidence gap. Guided by the Andersen behavioral model of health service use [8] and the health belief model [9], which emphasize predisposing, enabling, and perception-related factors influencing health-seeking behavior, this study aimed to assess the prevalence and determinants of vaccination dropout between BCG and measles vaccines among children aged 12 to 23 months in the Kikula Health Zone, Likasi, DRC.

## Methods

### Study Design and Setting

We conducted an analytical cross-sectional study between April 22 and May 22, 2025, in the Kikula Health Zone, Likasi city, Haut-Katanga province, DRC. The zone includes urban and periurban health areas with heterogeneous access to immunization services. The Kikula Health Zone was purposively selected due to its high vaccination dropout rate and the documented discrepancy between administrative coverage (>100%) and survey-based estimates (<30%).

### Study Population and Eligibility Criteria

The study population consisted of children aged 12 to 23 months and their mothers or primary caregivers.

### Inclusion Criteria

The inclusion criteria were as follows: children aged 12 to 23 months at the time of the survey, residence in the health zone for 6 months or more, presence of the mother or primary caregiver, and provision of written informed consent.

### Exclusion Criteria

The exclusion criteria were as follows: refusal to participate and children severely ill and unable to participate.

### Determination of Vaccination Status

Children with vaccination cards had their immunization status verified directly by card inspection, and children without vaccination cards were also included in this study. For these cases, vaccination status was determined through caregiver recall and, when available, verification against health facility registers. This approach ensured that all eligible children were represented in the study while acknowledging the potential for misclassification bias.

### Sampling Strategy

A three-stage cluster sampling technique was applied. First, health areas were selected using probability proportional to size. Second, streets or segments were randomly selected within each health area, with support from community health workers. Third, households were visited systematically (eg, every third household). If multiple eligible children were present, the youngest was selected to reduce recall bias. This approach ensured representativeness despite the absence of a complete sampling frame.

### Sample Size

Sample size was calculated using the Schwartz formula for cross-sectional studies:

n=Z^2^× *P*(1−*P*)/d^2^

where Z=1.96 (95% confidence level), *P*=.26 (dropout prevalence from previous Kikula surveys), and Cohen *d*=0.05 (margin of error).

The initial estimate (n=296) was adjusted for a design effect of 1.5 and rounded to 300 to account for nonresponse. In total, 322 households were approached, and 300 households completed the survey (response rate 93.2%).

### Recruitment Procedures

Prior to data collection, meetings were held with health zone authorities and community health workers to explain objectives and obtain authorization. Investigators introduced themselves at households, explained the study, verified eligibility, and obtained written consent. Noneligible or refusing households were skipped according to the sampling interval. Daily supervision ensured adherence to protocol and data quality.

### Data Collection

Data were collected using a structured, interviewer-administered questionnaire covering maternal sociodemographic characteristics (eg, age, education, marital status, occupation, religion, and duration of residence), child characteristics (eg, age, sex, and birth order), vaccination status verified by card, maternal knowledge (eg, importance of vaccination, schedule, vaccine-preventable diseases, and adverse events following immunization), perceptions of services (eg, reception, waiting time, information, vaccine availability, and perceived cost), and health system factors (eg, distance to the facility, participation in campaigns, and possession of a vaccination card).

The questionnaire was pretested in a nonselected health area. Investigators received training on ethical procedures, standardized administration, and card verification. Daily debriefings corrected inconsistencies.

### Variables

#### Dependent Variable

The dependent variable was vaccination dropout, defined as receipt of BCG but not the measles vaccine. The dropout rate was calculated using the following formula: dropout rate=(number of children who received BCG but not measles)/(number of children who received BCG)×100. The variable was coded as 1=dropout and 0=no dropout.

#### Independent Variables

The independent variables included the following: maternal sociodemographics (eg, age ≤25 y vs >25 y; education: none, primary, secondary, and higher; marital status; occupation; religion; and residence ≤5 y vs >5 y), child characteristics (eg, age, sex, and birth order), maternal knowledge (eg, importance, schedule, diseases, and adverse events), perceptions of services (eg, reception, waiting time, information, availability, and cost), and health system factors (eg, distance: <5 km, 5‐10 km, and >10 km; campaign exposure; and possession of a vaccination card).

#### Thresholds

Thresholds (eg, maternal age, distance, and residence duration) were based on regional literature and operational practice.

### Operational Definitions of Key Variables

Use of child health services was defined as whether the child had ever been taken to a health facility for preventive or curative services (eg, growth monitoring, vaccination, and consultation) in the past 12 months. “Yes” indicated the child received at least one service, while “no” indicated the child had not received any service.

Recourse to child health service was defined as the caregiver’s reported use of formal health services for the child (eg, vaccination sessions, consultations, and campaigns). “Yes” indicated that the caregiver reported attending at least one vaccination session or campaign, while “no” indicated that the caregiver reported never attending.

Possession of a vaccination card was defined as whether the caregiver could present the child’s vaccination card at the time of the survey. “Yes” indicated that the card was available and verified, while “no” indicated that the card was absent, and vaccination status was determined by recall and/or registers.

### Data Management

Incomplete questionnaires and cases without vaccination cards were excluded. Data were entered into Epi Info (version 7.2.3.1; Centers for Disease Control and Prevention), cleaned, and then exported to SPSS (version 26; IBM Corp). A partial double-entry procedure was performed to check consistency. No imputation was applied.

### Statistical Analysis

Data analysis was performed using descriptive, bivariate, and multivariate analyses:

- Descriptive analysis—frequencies and percentages were calculated for categorical variables, while means or medians were calculated for continuous variables.
- Bivariate analysis—chi-square or Fisher exact tests were used. Variables with *P*<.20 were retained for multivariate analysis.
- Multivariate analysis—logistic regression was performed to estimate adjusted odds ratios (AORs) with 95% CIs. Robust SEs were used to account for clustering. Multicollinearity was assessed using variance inflation factors. Statistical significance was set at *P*<.05.

Extremely high AORs (eg, >30,000) were interpreted cautiously, acknowledging that they likely reflect sparse data or near-perfect separation rather than precise effect sizes.

### Ethical Considerations

Ethics approval was obtained from the Ethics Committee of the School of Public Health, University of Kinshasa (ESP/CE/099/2023). Written informed consent was obtained from all participants. Data were anonymized, coded, and securely stored. No financial incentives were provided to participants, in accordance with national guidelines. The study results represented conservative estimates due to the exclusion of children without documented vaccination records.

## Results

### Overview

Among 300 children aged 12 to 23 months included in the survey, 115 (38.3%) had dropped out between BCG and measles vaccination, while 185 (61.7%) completed the schedule (Table 1).

Most respondents were mothers (295/300, 98.3%). Children were evenly distributed by sex (male: n=153, 51% and female: n=147, 49%). Most mothers were aged 25 years or younger (n=247, 82.3%). Education levels were no schooling (n=7, 2.3%), primary (n=70, 23.3%), secondary (n=207, 69.0%), and higher or university (n=16, 5.3%). Most mothers were housewives (n=230, 76.7%) and married (n=259, 86.3%). Protestant affiliation was predominant (n=164, 54.7%). Duration of residence exceeded 5 years for 174 (58%) mothers, while 126 (42%) had lived in the area for 5 years or less (Table 2).

**Table 1.** Vaccination status of children aged 12 to 23 months in the Kikula Health Zone, Likasi, Democratic Republic of Congo, April to May 2025 (cross-sectional survey).

Variables	Participants (N=300), n (%)
Vaccination abandonment	
Yes	115 (38.3)
No	185 (61.7)
Detention of vaccination card	
Yes	220 (73.3)
No	80 (26.7)
Respect for vaccination appointment	
Yes	201 (67.0)
No	99 (33.0)
Use children’s vaccines	
Yes	258 (86.0)
No	42 (14.0)

**Table 2.** Sociodemographic characteristics of mothers or caregivers and children aged 12 to 23 months in the Kikula Health Zone, Likasi, Democratic Republic of Congo, April to May 2025.

Variables	Effective (N=300), n (%)
Child’s guardian	
Others	5 (1.7)
Mother or father	295 (98.3)
Sex of the child	
Male	153 (51)
Female	147 (49)
Level of education	
No schooling	7 (2.3)
Primary	70 (23.3)
Secondary	207 (69.0)
Higher or university	16 (5.3)
Daily occupation	
Unemployed	5 (1.7)
Housewives	230 (76.7)
Workers	61 (20.3)
Self-employed	4 (1.3)
Marital status	
Single	17 (5.7)
Married	259 (86.3)
Widow	2 (0.7)
Divorced	22 (7.3)
Religion	
No religion	6 (2.0)
Catholic	95 (31.7)
Protestant	164 (54.7)
Muslim	3 (1.0)
Kimbanguist	11 (3.7)
Ancestral	21 (7.0)
Age (years)	
>25	53 (17.7)
≤25	247 (82.3)
Stays in the quarter or village (years)	
>5	174 (58.0)
≤5	126 (42.0)

Vaccination was considered “very important” by 103 (34.3%) of 300 mothers and “important” by 178 (59.3%) mothers. Only 8 (2.7%) mothers considered vaccination “not important.” More than half (n=169, 56.3%) of the mothers did not know their child’s vaccination status. Knowledge of adverse events following immunization was reported by 158 (52.7%) mothers, while 142 (47.3%) were unaware. Fear of side effects was expressed by 148 (49.3%) mothers. Nearly all households (n=289, 96.3%) reported low income. Concerns about vaccine safety for the child were expressed by 195 (65%) mothers (Table 3).

Reception at health facilities was rated “good” by 294 (98%) of 300 respondents. Waiting times were less than 1 hour for 163 (54.3%) respondents, 1 to 2 hours for 108 (36%), and more than 2 hours for 29 (9.7%). Information about vaccines was received by 234 (78%) respondents. Distance to facilities was less than 5 km for 223 (74.3%) respondents, 5 to 10 km for 54 (18%), and more than 10 km for 23 (7.7%). Most respondents (n=271, 90.3%) reported having received information about vaccination campaigns (Table 4).

**Table 3.** Maternal knowledge and attitudes toward vaccination among caregivers of children aged 12 to 23 months in the Kikula Health Zone, Likasi, Democratic Republic of Congo, April to May 2025.

Variables	Effective (N=300), n (%)
Importance of vaccination	
Very important	103 (34.3)
Important	178 (59.3)
Not important	8 (2.7)
Moderately important	11 (3.7)
Knowledge of who administers vaccines	
Failing	272 (90.7)
Acceptable	24 (8.0)
Good	4 (1.3)
Child’s vaccination status	
Does not know	169 (56.3)
Knows	131 (43.7)
Knowledge of adverse events following immunization	
Does not know	142 (47.3)
Knows	158 (52.7)
Fear of side effects	
No	152 (50.7)
Yes	148 (49.3)
Cost of living for mothers or child care providers	
Lower	289 (96.3)
Higher	11 (3.7)
Concern about the vaccine for the baby	
No	105 (35.0)
Yes	195 (65.0)

**Table 4.** Perceptions of immunization services among caregivers of children aged 12 to 23 months in the Kikula Health Zone, Likasi, Democratic Republic of Congo, April to May 2025.

Variables	Effective (N=300), n (%)
Reception at health facility	
Bad	6 (2.0)
Good	294 (98.0)
Waiting time (hours)	
<1	163 (54.3)
1‐2	108 (36.0)
>2	29 (9.7)
Information about the nature of the vaccine used	
No	66 (22.0)
Yes	234 (78.0)
Distance from the house to the vaccination service (km)	
<5	223 (74.3)
5‐10	54 (18.0)
>10	23 (7.7)
Perceived cost of vaccination	
Free	263 (87.7)
Moderate	35 (11.7)
High	2 (0.6)
Information received about vaccination campaigns	
No	29 (9.7)
Yes	271 (90.3)

### Bivariate Analysis

Dropout was significantly associated with the following: duration of residence of 5 years or less (odds ratio [OR] 1.95, 95% CI 1.20‐3.72; *P*=.007), nonuse of child health services (OR 4.62, 95% CI 2.20‐9.01; *P*<.001), absence of a vaccination card (OR 44.19, 95% CI 18.98‐102.91; *P*<.001), and lower maternal education (no schooling: OR 42.0, 95% CI 3.16‐556.5; *P*=.001 and primary education: OR 7.41, 95% CI 1.56‐35.1; *P*=.001; Table 5).

**Table 5.** Factors associated with vaccination dropout between Bacillus Calmette-Guérin and measles vaccines among children aged 12 to 23 months in the Kikula Health Zone, Likasi, Democratic Republic of Congo, April to May 2025 (bivariate and multivariate logistic regression).

Variables	Vaccination abandonment		Bivariate analysis (chi-square)		Multivariate analysis	
	Yes (n=115), n (%)	No (n=185), n (%)	OR^a^ (95% CI)	*P* value	Adjusted OR (95% CI)	*P* value
Age group (years)						
>25	94 (81.7)	153 (82.7)	0.9 (0.51-1.72)	.83	1.0 (0.4-2.5)	.92
≤25	21 (18.3)	32 (17.3)	1^b^	—^c^	1	—
Stays in the quarter or village (years)						
>5	37 (32.2)	89 (48.1)	1	—	1	—
≤5	78 (67.8)	96 (51.9)	2.0 (1.20-3.72)	.007	1.6 (0.83-3.28)	.16
Use children’s vaccines						
No	29 (25.2)	13 (7.0)	4.6 (2.20-9.01)	.001	1.0 (0.38-2.83)	.94
Yes	86 (74.8)	172 (93.0)	1	—	1	—
Possession of a child health or vaccination card						
No	73 (63.5)	7 (3.8)	44.2 (18.98-102.91)	<.001	30.9 (11.6-82.0)	<.001
Yes	42 (36.5)	178 (96.2)	1	—	1	—
Educational level						
No schooling	6 (5.2)	1 (0.5)	42.0 (3.16-556.50)	<.001	3.7 (0.10-142.17)	.48
Primary	36 (31.3)	34 (18.4)	7.4 (1.56-35.06)	.001	2.6 (0.26-24.98)	.42
Secondary	71 (61.7)	136 (73.5)	3.7 (0.80-16.56)	.07	3.7 (0.43-31.93)	.24
Higher or university	2 (1.7)	14 (7.6)	1	—	1	—
Daily occupation						
Unemployed	3 (2.6)	2 (1.1)	1	—	1	—
Housewives	99 (86.1)	131 (70.8)	0.5 (0.08-3.07)	.45	3.1 (0.11-83.13)	.50
Workers	10 (8.7)	51 (27.6)	0.1 (0.02-0.88)	.02	4.0 (0.51-31.82)	.19
Self-employed	39 (33.9)	1 (0.5)	26.0 (1.79-376.32)	.001	10.8 (1.09-106.84)	.04
Marital status						
Single	4 (3.5)	13 (7.0)	1	—	1	—
Married	96 (83.5)	163 (88.1)	1.9 (0.60-6.03)	.26	1.2 (0.17-8.43)	.86
Widow	2 (1.7)	0 (0.0)	ind^d^	—	—	—
Divorced	13 (11.3)	9 (4.9)	4.7 (1.15-19.16)	.03	1.0 (0.26-4.08)	.97
Religion						
No religion	5 (4.3)	1 (0.5)	2.5 (0.10-62.60)	—	1.4 (0.03-77.83)	.86
Catholic	23 (20.0)	72 (38.9)	0.1 (0.01-1.84)	—	3.5 (0.20-63.42)	.39
Protestant	72 (62.6)	92 (49.7)	0.4 (0.03-4.40)	—	2.2 (0.13-38.39)	.57
Muslim	2 (1.7)	1 (0.5)	1	—	4.0 (0.13-116.66)	.43
Kimbanguist	4 (3.5)	7 (3.8)	0.3 (0.01-4.23)	—	4.3 (0.20-92.66)	.35
Ancestral	9 (7.8)	12 (6.5)	0.4 (0.02-4.80)	.005	1.4 (0.03-77.83)	.86
Importance of vaccination						
Very important	39 (33.9)	64 (34.6)	1	—	—	—
Important	61 (53.0)	117 (63.2)	0.9 (0.51-1.41)	—	0.9 (0.42-1.81)	.73
Not important	6 (5.2)	2 (1.1)	4.9 (0.94-25.61)	—	0.6 (0.03-10.30)	.72
Moderately important	9 (7.8)	2 (1.1)	7.4 (1.51-35.96)	.005	2.5 (0.28-22.12)	.41
Knowledge of who administers vaccines						
Failing	111 (96.5)	161 (87.0)	0.2 (0.02-2.23)	—	3.8 (0.26‐56.46)	.33
Acceptable	1 (0.9)	23 (12.4)	0.0 (0.00-0.29)	—	29.6 (1.29‐680.31)	.03
Good	3 (2.6)	1 (0.5)	1	—	—	—
Child’s vaccination status						
Does not know	82 (71.3)	87 (47.0)	2.8 (1.70-4.59)	—	1.7 (0.74-4.05)	.21
Knows	33 (28.7)	98 (53.0)	—	—	—	—
Knowledge of adverse events following immunization						
Does not know	78 (67.8)	64 (34.6)	4.0 (2.42-6.53)	—	1.3 (0.54-3.26)	.53
Knows	37 (32.2)	121 (65.4)	—	—	—	—
Fear of side effects						
No	74 (64.3)	78 (42.2)	2.5 (1.53-4.00)	—	1.4 (0.66-2.82)	.39
Yes	41 (35.7)	107 (57.8)	—	—	—	—
Waiting time (hours)						
<1	82 (71.3)	81 (43.8)	1	—	—	—
1-2	24 (20.9)	84 (45.4)	0.3 (0.1-0.48)	—	0.9 (0.30-2.48)	.80
>2	9 (7.8)	20 (10.8)	0.4 (0.19-1.03)	—	1.3 (0.46-3.78)	.59
Distance from the house to the vaccination service (km)						
<5	82 (71.3)	141 (76.2)	1	—	1.0 (0.265-3.870)	.99
5‐10	21 (18.3)	33 (17.8)	1.1 (0.59-2.01)	—	1.0 (0.265-3.870)	.99
>10	12 (10.4)	11 (5.9)	1.9 (0.79-4.44)	—	0.9 (0.210-3.829)	.88

a OR: odds ratio.

b OR=1 indicates the reference category.

c Not applicable.

d ind: indeterminate (n value too small).

### Multivariate Analysis

After adjustment, only possession of a vaccination card remained a strong independent predictor of dropout: children without a vaccination card had an AOR of 30.9 (95% CI 11.6‐82.0; *P*<.001).

Extremely high ORs and wide CIs should be interpreted with caution. These values likely reflect sparse data or near-perfect separation rather than precise effect sizes.

## Discussion

### Principal Findings

This analytic cross-sectional study of 300 mother-child pairs in the Kikula Health Zone revealed a validated vaccination dropout rate of 38.3% between BCG and measles vaccines. Possession of a child health or vaccination card was the strongest independent predictor of dropout, with children lacking cards having nearly 31-fold higher odds of incomplete immunization. Other factors, such as shorter duration of residence, lower maternal education, and nonuse of child health services, were significant in bivariate analysis but lost significance in multivariate models, likely due to collinearity and small subgroup sizes. These findings highlight the critical role of vaccination documentation and maternal knowledge in sustaining immunization coverage.

### Interpretation in Light of Theoretical Frameworks

The results can be interpreted through the Andersen behavioral model of health service use [8] and the health belief model [9]. Predisposing factors such as maternal age, education, and marital status influenced vaccination behavior, although their effects diminished after adjustment. Enabling factors such as possession of a vaccination card and use of child health services facilitated completion of the schedule. Perceptions such as maternal fear of side effects and lack of knowledge about vaccination status contributed to dropout. These frameworks underscore that vaccination behavior is shaped not only by access but also by perceptions and enabling resources.

Perceptions—maternal fear of side effects and lack of knowledge about vaccination status contributed to dropout.

### Comparison With Previous Studies

Our findings are consistent with national surveys (MICS 2017‐2018) reporting low full immunization coverage (<40%) and high dropout rates in the DRC [6,7]. These findings highlight the critical role of vaccination documentation and maternal knowledge in sustaining immunization coverage. Similar associations between maternal education, vaccination documentation, and service use have been reported in other sub-Saharan African settings [5]. However, the magnitude of the effect of card possession in this study (AOR 30.9, 95% CI 11.6‐82.0) is unusually high, reflecting the centrality of documentation in this context. Extremely high ORs and wide CIs should be interpreted with caution, as they likely reflect sparse data or near-perfect separation rather than precise effect sizes [12].

### Implications for Programmatic Action

The discrepancy between administrative coverage (>100%) and survey-based findings (38.3% dropout) highlights systemic weaknesses in data quality [10,11]. Inflated administrative reports may delay corrective measures and misguide resource allocation. Programmatic priorities should include strengthening card management and documentation systems, enhancing maternal education and awareness of vaccination schedules, deploying mobile vaccination units to reach underserved households, improving communication strategies to address fears of side effects, and integrating dropout monitoring into routine supervision to ensure timely corrective action.

These measures are essential to reduce dropout and improve equity in immunization coverage in the DRC.

### Limitations

This study has several limitations. First, the inclusion of children without vaccination cards, whose immunization status was determined through caregiver recall and health facility registers, may have introduced misclassification bias. Therefore, the results should be interpreted with caution, although this approach allowed for a more comprehensive assessment of dropout determinants. Second, the cross-sectional design precludes causal inference. Third, some variables lost statistical significance in multivariate analysis due to collinearity and small subgroup sizes, which limits precision. Fourth, the study was conducted in a single health zone, which may restrict generalizability to other settings. Despite these limitations, the sample size (N=300) was adequate to detect significant associations, and the findings provide valuable insights for programmatic action.

### Conclusions

Vaccination dropout between BCG and measles remains high in the Kikula Health Zone, driven primarily by lack of documentation and maternal knowledge gaps. Addressing these barriers through improved card management, maternal education, and targeted outreach is critical to strengthening immunization coverage. The findings underscore the need for accurate data, integrated monitoring, and context-adapted interventions to reduce dropout and achieve equitable immunization in the DRC.

## Abbreviations

AOR: adjusted odds ratio
BCG: Bacillus Calmette-Guérin
DRC: Democratic Republic of Congo
DTP3: third dose of the diphtheria-tetanus-pertussis vaccine
MCV1: first dose of measles-containing vaccine
MICS: Multiple Indicator Cluster Survey
OR: odds ratio
WHO: World Health Organization
